# Phylo2Vec: A Vector Representation for Binary Trees

**DOI:** 10.1093/sysbio/syae030

**Published:** 2024-06-27

**Authors:** Matthew J Penn, Neil Scheidwasser, Mark P Khurana, David A Duchêne, Christl A Donnelly, Samir Bhatt

**Affiliations:** Department of Statistics, University of Oxford, 24-29 St Giles’, Oxford OX1 3LB, UK; Department of Public Health, Section of Epidemiology, University of Copenhagen, Øster Farimagsgade 5, build. 24 Q, 1st floor, 1353 København K, Denmark; Department of Public Health, Section of Epidemiology, University of Copenhagen, Øster Farimagsgade 5, build. 24 Q, 1st floor, 1353 København K, Denmark; Department of Public Health, Section of Epidemiology, University of Copenhagen, Øster Farimagsgade 5, build. 24 Q, 1st floor, 1353 København K, Denmark; Department of Statistics, University of Oxford, 24-29 St Giles’, Oxford OX1 3LB, UK; Nuffield Department of Medicine, Pandemic Sciences Institute, University of Oxford, Old Road Campus Research Building, Old Road Campus, Roosevelt Drive, Oxford OX3 7DQ, UK; Department of Public Health, Section of Epidemiology, University of Copenhagen, Øster Farimagsgade 5, build. 24 Q, 1st floor, 1353 København K, Denmark; Department of Infectious Disease Epidemiology, School of Public Health, Faculty of Medicine, MRC Centre for Global Infectious Disease Analysis, Imperial College London, Level 2, Faculty Building, South Kensington Campus, London SW7 2AZ, UK

**Keywords:** Binary trees, optimization, phylogenetics, representation

## Abstract

Binary phylogenetic trees inferred from biological data are central to understanding the shared history among evolutionary units. However, inferring the placement of latent nodes in a tree is computationally expensive. State-of-the-art methods rely on carefully designed heuristics for tree search, using different data structures for easy manipulation (e.g., classes in object-oriented programming languages) and readable representation of trees (e.g., Newick-format strings). Here, we present Phylo2Vec, a parsimonious encoding for phylogenetic trees that serves as a unified approach for both manipulating and representing phylogenetic trees. Phylo2Vec maps any binary tree with n leaves to a unique integer vector of length n-1. The advantages of Phylo2Vec are 4-fold: (i) fast tree sampling, (ii) compressed tree representation compared to a Newick string, (iii) quick and unambiguous verification if 2 binary trees are identical topologically, and (iv) systematic ability to traverse tree space in very large or small jumps. As a proof of concept, we use Phylo2Vec for ML inference on 5 real-world datasets and show that a simple hill-climbing-based optimization scheme can efficiently traverse the vastness of tree space from a random to an optimal tree.

Phylogenetic trees are a fundamental tool for depicting evolutionary processes, whether linguistic (evolution of different languages and language families) or biological (evolution of biological entities). Within the biological sciences, phylogenetic trees are integral to multiple research domains, including evolution (Morlon et al. 2010), conservation (Rolland et al. 2011), and epidemiology, where they allow us to better understand infectious disease transmission dynamics (Ypma et al. 2013; Faria et al. 2021).

A multitude of computer-readable formats have been proposed to store and represent (binary) phylogenetic trees. Although basic data structures, such as arrays or linked lists, can be used for this purpose, the Newick format, as outlined by Olsen (1990) and Felsenstein (2004), has emerged as the standard notation. This format characterizes a tree through a string of nested parentheses. Each parenthesis encloses a pair of leaf nodes or subtrees separated by a comma. Additional metadata such as branch lengths can be added after a colon that follows the leaf node or subtree. Although it is a compact and intuitive notation, several limitations exist. First, comparing large trees using the Newick format can be difficult for human readers, especially as isomorphic trees can be obtained by permuting nodes or subtrees within a set of parentheses. Second, verifying that two trees are identical from Newick strings requires additional steps, as two identical trees need not have the identical Newick string. Alternative, bijective representations do exist. For example, several methods have been proposed to assign unique integers to binary trees with unlabeled (Rotem and Varol 1978; Proskurowski 1980), fully labeled (Knuth 1973), and partially labeled nodes (Rohlf 1983) (only leaf nodes). More recently, Palacios et al. (2022) investigated several enumeration strategies of binary trees compatible with a perfect phylogeny. However, as mentioned by Rohlf (1983), using single-integer representations for downstream phylogenetic analyses is computationally difficult for large trees due to the factorial rate at which the size of binary tree space grows (Cavalli-Sforza and Edwards 1967). Conversely, several vector representations such as graph polynomials (Liu 2021; Liu et al. 2022) and the compact bijective ladderized vector (Voznica et al. 2022) were introduced as a support for model selection and estimation of evolutionary or epidemiological parameters. Other vector representations of tree topology, such as pair matchings (Diaconis and Holmes 1998) and F matrices (Kim et al. 2020), focus on the polynomial-time computation of the distance between any 2 trees (to measure similarity). However, methods for systematically sampling random trees or changing tree topology with respect to an objective function by leveraging such vector representations have been understudied. In particular, creating sampling schemes (as done in Bayesian frameworks such as BEAST (Drummond and Rambaut 2007; Bouckaert et al. 2014) and MrBayes (Huelsenbeck and Ronquist 2001)) around standard tree arrangements is non-trivial, and, although inferring phylogenetic trees is a common task in evolutionary biology, tree search using any optimality criteria (including ML) is NP-hard (Roch 2006). Another critical challenge is the size of the tree space: for a tree with *n* leaves, there are (2n-3)⋅(2n-5)⋅…⋅5⋅3⋅1 possible rooted binary trees (Cavalli-Sforza and Edwards 1967). Finally, optimization-based approaches often face a jagged “loss” landscape containing many trees with the same criterion score (Sanderson et al. 2011). When considering inference, the choice of representation can be particularly relevant for application to real phylogenetic problems. For example, an application of the approach we introduce here can be used for continuous relaxation and gradient descent under the minimum evolution criterion (Penn et al. 2023). For large phylogenies, the use of an efficient representation such as the compact bijective ladderized vector (Voznica et al. 2022) has proven effective for deep learning-based, likelihood-free, inference (Thompson et al. 2024), or diversification inference (Lambert et al. 2023).

To overcome these limitations, we introduce Phylo2Vec, a new representation for any binary tree. In this framework, the topology of a binary tree can be completely described by a single integer vector v of dimension n-1, where n is the number of leaves in the tree. The vector’s construction is intrinsically related to the branching pattern of the tree, and is defined by a simple constraint: $v_{j}\in \{0,1,\ldots ,2(j-1)\}$ for all j∈{1,…,n-1}. The approach we present here is most similar to that previously introduced by Rohlf (1983), but we focus on the integer representation and its mathematical properties, rather than counting or labeling trees. Additionally, this formulation naturally offers a new measure of distance between trees (e.g., by comparing 2 vectors using the Hamming distance) and yields a new mechanism to explore tree space that diverges from traditional heuristics such as subtree, prune, and regraft (SPR). To further demonstrate its utility, as a proof of concept, we apply Phylo2Vec to several phylogenetic inference problems, where the task is to find an optimal tree given a set of genetic sequences using maximum likelihood (ML) estimation. While state-of-the-art frameworks for phylogenetic inference typically rely on search heuristics based on deterministic tree arrangements, Phylo2Vec provides the first steps to a more systematic criterion for optimization.

## Materials and Methods

The goal of this project was to develop a bijection (i.e., a one-to-one correspondence) between the set of binary rooted trees with n leaves to a constrained set of integer vectors of length n-1. We first describe an intuitive but incomplete (as not bijective) integer representation of trees as birth processes. Second, we define and characterize Phylo2Vec as a bijective generalization of this first representation and formalize its properties. Third, we showcase the utility of Phylo2Vec by applying the representation for MLE-based phylogenetic inference on empirical datasets.

Our construction draws from an existing method of assigning integer counts to trees (Rohlf 1983), although we focus on vector representations. It is distinct from Rohlf (1983) in labeling the tree edges, motivated by a simple and intuitive representation of birth processes. By applying this encoding to rooted binary trees, we are able to move around tree space similarly to subtree-prune and regraft methods. Furthermore, we provide a rigorous proof of its bijectivity alongside a range of algorithms (all implemented into a Python package) that allows researchers to build on the phylogenetic optimization algorithm we present here. Thus, we provide a significantly different method from those proposed previously (Rohlf 1983), by focusing our efforts toward practical transitions in tree space.

### An Incomplete Integer Representation of Trees as Birth Processes

Let T denote a rooted phylogenetic tree with n leaf nodes representing (biological) taxa, and D symbolize a key-value mapping (or dictionary) that associates a nonnegative integer (the keys) to each leaf node (the values).

Using this mapping, *for a subset of all trees*, we can summarize their topology using an integer vector v of size n-1 such that:


$$\begin{array}{c} v_{j}\in \{0,1,\ldots ,j-1\}\forall j\in \{1,\ldots ,n-1\} \end{array}$$ (1)

**Figure 1:**
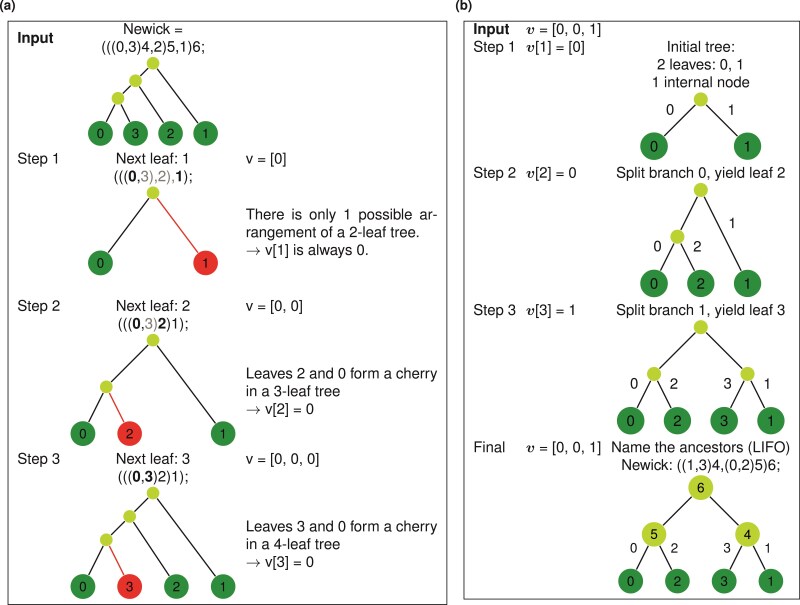
An incomplete integer representation of tree topology as birth processes. a) Labeling a tree as an ordered vector: example for v=[0,0,0]. We process leaves in ascending order. For each leaf j, we retrieve its sibling (or adjacent tip) in the Newick string, ignoring leaves > j. The adjacent tip corresponds to v[j]. b) Recovering a tree from an ordered vector: example for v=[0,0,1]. We process v from left to right. Ancestors are named in last-in-first-out (LIFO) fashion: The ancestor of the last added leaf L-1 (here, leaf 3) is named L (here, 4), the ancestor of the second-to-last added leaf L-2 (here, leaf 2) is named L+1 (here, 5), and so on. In both cases, the lengths of the edges are arbitrary.

The construction of this vector is inspired by birth processes: assuming a two-leaf tree with leaves labeled 0 and 1, we process v from left to right. For each j∈{1,…,n-1}, $v_{j}$ (hereinafter noted as v[j]) denotes the addition of leaf j such that, at iteration j, leaf j forms a cherry with leaf v[j]. In other words, the branch leading to leaf v[j] “gives birth” to leaf j. Figure 1 illustrates algorithms to convert a tree to a vector and *vice versa*.

Although a simple representation of tree topology, it is easy to see from Equation (1) that this construction is incomplete. Indeed, there are j possible values for any v[j], and thus, for n leaves, there are 1⋅2⋅…⋅(n-1)=(n-1)! possible vectors, which is less than the number of binary rooted trees, (2n-3)!! (where !! denotes the semifactorial) (Cavalli-Sforza and Edwards 1967; Felsenstein 1978; Diaconis and Holmes 1998). This discrepancy stems from the assumptions of this construction, whereby a new leaf j has to form a cherry with a previously processed leaf 0,1,…,j-1. For instance, leaf 2 has to form a cherry with either leaf 0 or 1, but cannot be an outgroup of the (0, 1) subtree. We thus denote trees that follow this incomplete construction of tree space as “ordered” trees, as they require a precise ordering of the leaf nodes.

### Phylo2Vec

In this section, we define and formalize the properties of Phylo2Vec, an integer vector representation that extends the formulation presented above to be valid for any rooted binary tree. To ensure bijectivity to this space, we need the vector v to satisfy the following constraints:


$$\begin{array}{c} v_{j}\in \{0,1,\ldots ,2(j-1)\}\forall j\in \{1,\ldots ,n-1\}. \end{array}$$ (2)

We say v∈V if Equation (2) is satisfied. For this representation, there are 2j-1 entries for any position j. Therefore, the number of possible vectors matches the number of possible binary-rooted trees:


$$\overset{n-1}{\underset{j=1}{\prod }}(2j-1)=(2(n-1)-1)!!=(2n-3)!!$$

From this observation, we can prove the bijectivity of the mapping simply by showing injectivity—that is, that no two distinct vectors v and w lead to the same tree. A proof is presented in the Appendix (Phylo2Vec details). Briefly, our proof relies on the fact that certain properties of pairs of nodes are preserved throughout the construction process—namely, that the most recent common ancestor (MRCA) of a pair of nodes is unchanged (once both nodes have been added to the tree) and that if one node is the ancestor of another at some stage of the construction process, then this remains true in the final tree. Then, if T and $T^{\prime }$ are the trees resulting from different vectors v and v′, respectively, we choose the smallest i such that $v_{i}\neq v_{i}^{\prime }$. By considering the sets of leaf nodes descended from the edge to which i is added, we can show that the addition of node i causes a pair of nodes to either have a different MRCA or a different ancestral relationship. Therefore, since these properties are preserved throughout the construction process, we must have $T\neq T^{\prime }$. This shows the injectivity of our mapping, with bijectivity following from the fact that the number of trees is the same as the number of possible vectors v.

#### Recovering a tree from a Phylo2Vec vector

**Figure 2:**
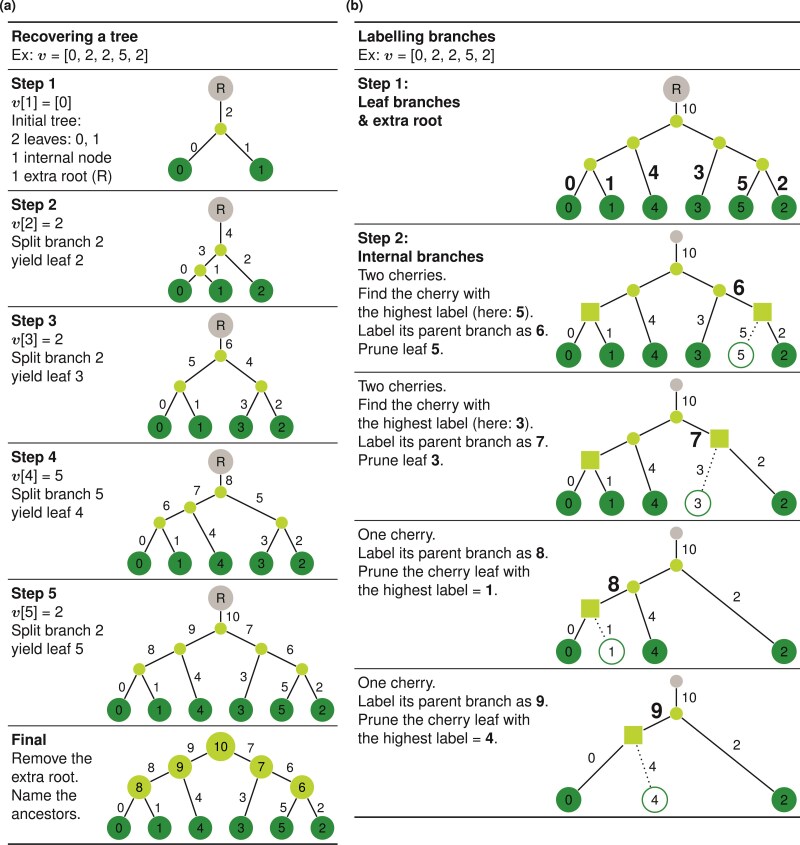
Recovering a tree from a Phylo2Vec vector: example for v=[0,2,2,5,2]. a) Main algorithm for leaf placement. Initially, we consider a tree with 2 leaves labeled 0 and 1 and an extra temporary root, which ensures that there are 2j-1 entries for any position j∈{1,…,n-1}. This state corresponds to v= [0]. We then process v from left to right. v[j] denotes the branch to be split, yielding a new leaf j. At the end of each iteration, a branch labeling step described in b) is performed. First, branches leading to leaves 0, ..., n-1 are labeled 0, ..., n-1, respectively. Second, the temporary root is always labeled as 2(n-1). Third, for internal branches, the next branch (n) to label is the branch of a cherry with the highest label $c_{max}$. We then prune out the leaf $c_{max}$ and repeat the same process for internal branches n+1,...,2(n-1)-1. See Supplementary Fig.S2a for more details about complexity.

Building a binary tree from v follows closely the algorithm in Figure 1, but incorporates two additional requirements. First, we start from a two-leaf tree, whereby the leaves are labeled 0 and 1. The branches that lead to leaves 0, 1 are also labeled 0, 1, respectively. Second, we draw an additional node (called the “extra root”) which is initially connected to the root by a branch labeled 2 (see second row in Fig. 2).

**Figure 3:**
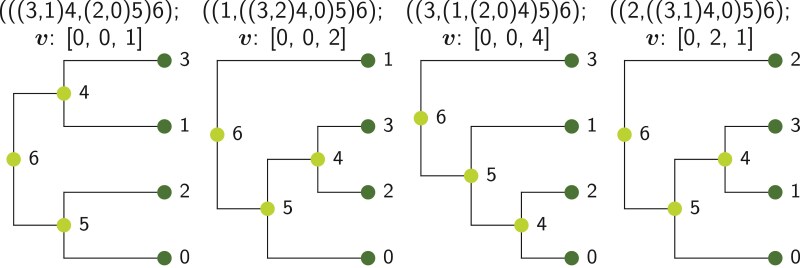
Example of trees with n=4 leaves represented in both Newick and Phylo2Vec vector formats. Nodes 0-3 and 4-6, respectively, denote the leaves and internal nodes.

**Figure 4:**
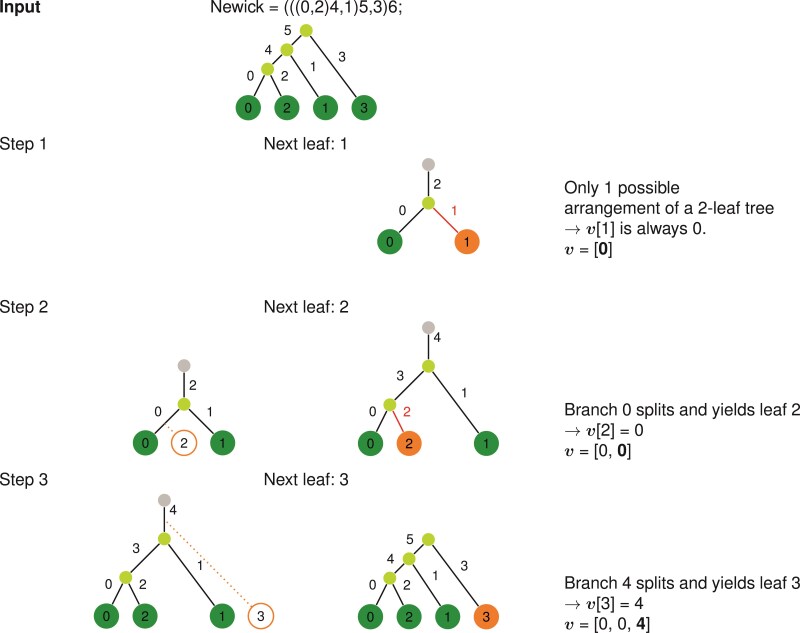
Labeling a tree as a Phylo2Vec vector v : example for v= [0, 0, 4]. We process leaves in ascending order. For each leaf j, we determine the branch that split and yielded leaf j, which corresponds to v[j]. At each step, we re-label the branches with the same process as in Figure 2.

The addition of a temporary root in the construction of v from a tree and vice versa ensures that there are 2j-1 branches from which a leaf j can descend from. From these requirements, we can build a unique rooted tree T by processing v from left to right, where v[j] indicates the branch that will split and yield leaf j. Figure 2 shows a detailed example of this scheme, and other example representations for trees with n=4 leaves are shown in Figure 3. We also describe (and prove its existence in the Appendix) an inverse algorithm to convert a tree represented in Newick format as a Phylo2Vec vector in Figure 4. To ensure consistency when converting a v to a Newick string and *vice versa*, we also describe in Figure 2b an algorithm to label branches based on iterative cherry-picking operations.

#### Complexity

The algorithm underlying Figure 2 and detailed in Algorithm S3 has an average time complexity of $O(n^{1.5})$ and a worst-case time complexity of $O(n^{2})$ due to the linear cost insert step. However, an implementation using a data structure based on Adelson-Velsky and Landis (AVL) trees is possible that would allow Algorithm S3 to run in linearithmic time (O(nlog ⁡n); an implementation is described in (Scheidwasser and Jakob 2024)). This self-balancing tree implementation is only faster for large trees (over ≈50,000 taxa). A basic version using NumPy (Harris et al. 2020) runs in a few milliseconds for n=10,00 taxa on a modern CPU. The inverse algorithm (converting a Newick string to a Phylo2Vec v), detailed in Algorithm S4, is also of linearithmic complexity when internal nodes are already labeled (according to the scheme described in Fig. 2) and quadratic otherwise (see Fig.S2b). Speedups are made through just-in-time compilation, for example, using Numba (Lam et al. 2015) in Python.

#### Distances between trees

The formulation of Phylo2Vec as a one-to-one correspondence between binary trees and integer vectors constrained by Equation (2) naturally allows for a new measure of distance between trees. For any two Phylo2Vec trees v and w, a Hamming distance can be defined as


$$\begin{array}{c} \mu (v,w)=\overset{n-1}{\underset{i=1}{\sum }}I_{v_{i}\neq w_{i}}. \end{array}$$ (3)

To compare this distance with other tree distance metrics, we consider a simple discrete random walk in the space of possible Phylo2Vec vectors V. At each step, we create a new vector w from the previous vector v as follows. First, we choose a random subset of the indices I⊆{2…,n-2}. For each i∉I, we set $w_{i}=v_{i}$ and for each i∈I, $w_{i}=\min(2(i-1),\max(v_{i}+J(i),0))$ where the J(i) are iid random variables uniform on the set {-1,1}. Note that the values of J(i) at different steps of the walk are also independent, and that the minimum and maximum in the definition of $w_{i}$ ensure that it satisfies the constraint $0\leq w_{i}\leq 2(i-1)$.

**Figure 5:**
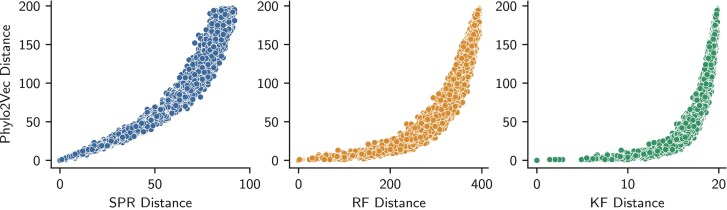
Comparison of Phylo2Vec moves with 3 popular tree distances: subtree-prune-and-regraft (SPR; left), Robinson–Foulds (RF; middle), and Kuhner–Felsenstein (KF; right). To generate the distances, a random walk of 5000 steps was performed from a random initial v with 200 taxa. At each step, each $v_{i}$ can increment, decrement, or remain unchanged.

As 1,n-1∉I, we fix $w_{1}=0$ (by our constraints) and $w_{n-1}=2(n-2)$ (to ensure that we move in the unrooted tree space for SPR distance calculations). Figure 5 compares μ to an approximate SPR distance (De Oliveira Martins et al. 2016), Robinson–Foulds (RF) distance (Robinson and Foulds 1981), and Kuhner–Felsenstein (KF) distance (Kuhner and Felsenstein 1994). We note that exact, rooted distance for SPR is nondeterministic polynomial-time hard (NP-hard) to compute (Bordewich and Semple 2005) and, therefore, cannot be directly compared to our rooted Phylo2Vec formulation. For all distances, we see a nonlinear correspondence, especially for RF and KF distance. Small changes in v can lead to very large topological jumps, but equally, small jumps are also possible. Modifying several indexes in v results in significant jumps across tree space, leading to new trees that are very dissimilar. As a result, SPR, RF, or KF distances saturate as we increase the number of changes in v (St. John 2017). However, we note that small changes in $v_{i}$ can also readily correspond to very minor topological changes.

In the exploration of tree space, the number of possible moves for both SPR and Phylo2Vec is of order $O(n^{2})$ (see Phylo2Vec details in the Appendix). Consequently, Phylo2Vec is expected to explore tree space in a similar manner than SPR, with proposals being less local than nearest neighbor interchange but also less global than those by tree bisection reconnection. However, the number of *single* SPR changes is approximately 4 times greater than the number of *single* changes in v(i.e., changes of a single index of v) as SPR considers internal nodes while v is defined across the leaves. Thus, Phylo2Vec changes are likely a subset of possible SPR changes.

Whereas Figure 5 shows distances between unrooted trees, our framework is built on rooted phylogenies at its core. Knowing that all rootings produce the same likelihood due to the pulley principle and reversibility of nucleotide substitution models (Felsenstein 2004), we can, for any rooted phylogeny, switch to one that is rooted at a different outgroup and has exactly the same likelihood. Thus, an equivalence class V exists where, given a likelihood or parsimony score ℓ, any given Phylo2Vec vector v∈V has the same ℓ(v), an SPR or RF distance of 0, but a Phylo2Vec distance of μ>0. In practice, μ is often very large between v∈V (comparable to half the maximum SPR distance, see Supplementary Fig.S1), which makes switching a vector v∈V to an equivalent v′∈V an additional mechanism for tree space exploration.

#### Shuffling indices

**Figure 6:**
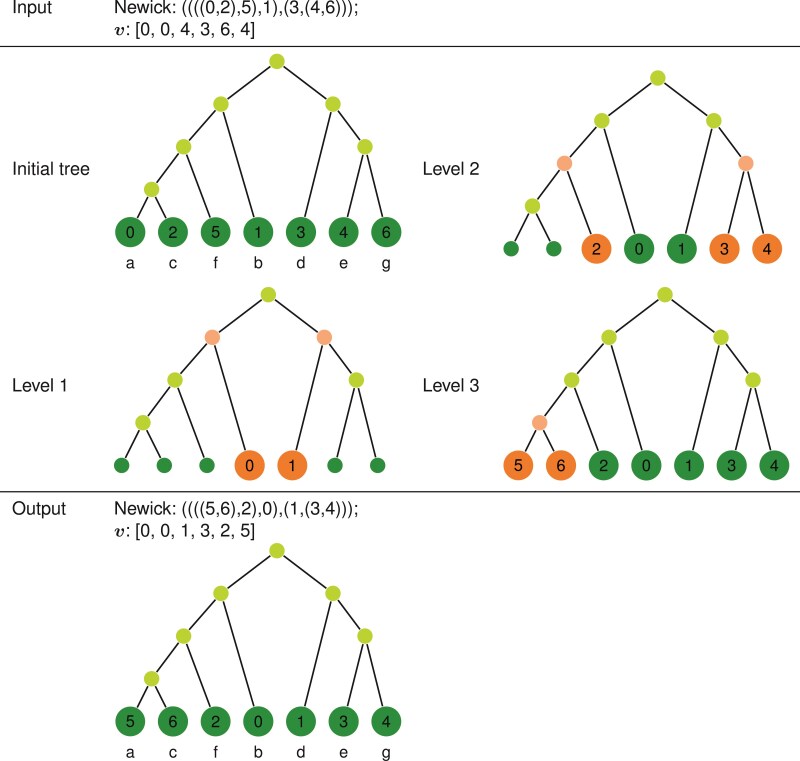
Example of a reordering scheme of v using level-order traversal. Starting from the root, for each level, we relabel the immediately descending leaf nodes with the smallest integers available (from 0 to n-1). The letters (a–g) indicate the taxa, showing that reordering the leaves does not affect tree topology but simply changes the integer-taxon mapping.

This distance between two trees is not symmetric with respect to the labeling of the trees, as discussed further in the Appendix (Phylo2Vec details). Depending on the choice of labeling, certain portions of the tree may be easier to optimize than others when performing phylogenetic inference. This is an undesirable quality and can be remedied by a simple reordering of indices within our algorithm. An example of a reordering algorithm is presented in Figure 6.

Consider a tree T where the leaves are labeled by a fixed set of indices {1,2,…,n-1}. Suppose that σ is a permutation of {1,2,…,n-1}, and consider a shuffled tree σ(T) with the same topological structure as T, but where, for each j∈{1,2,…,n-1}, the leaf with original label i now has label σ(j).

Calculating the likelihood requires a tree T and a set of genetic data $D=(D_{1},D_{2},\ldots ,D_{n-1})$, where $D_{j}$ corresponds to the physical or genetic characteristics of leaf j. We can then write the likelihood as L=L(T,D). Moreover, defining the shuffled genetic data as $\sigma (D)=(D_{\sigma^{-1}(1)},D_{\sigma^{-1}(2)},\ldots ,D_{\sigma^{-1}(n-1)})$, we can then see that L(T,D)=L(σ(T),σ(D)). This occurs because when computing the likelihood, any calculation for L(T,D) that involves the node with original label i (and hence genetic data $D_{i}$) will now involve the node with label σ(i) and hence genetic data $D_{\sigma^{-1}(\sigma (i))}=D_{i}$. Should the permutation only be applied to either the tree labels or the genetic data set, the resulting likelihood will likely be different from L(T,D). Thus, since the topological structure of T is the same as D(T), the likelihood will remain unchanged. A more rigorous proof can be found in the Appendix (Phylo2Vec details).

One can also recover the vector v corresponding to the shuffled tree σ(T). This is possible because of the bijective relationship between the space of v’s and the space of trees. We provide an algorithm that inverts our map from v to M in the Appendix (Phylo2Vec details). Thus, one can equivalently define a shuffled vector σ(v) (such that σ(v) generates σ(T)) and consider the likelihood relationship as L(v,D)=L(σ(v),σ(D)). This allows for discrete optimization steps to be taken with respect to the new shuffled v, increasing the flexibility of the algorithm while removing the asymmetric effects of the initial labeling.

#### Branch lengths

In addition to tree topology, determining the branch lengths of a tree is an important facet in phylogenetic inference. When making small changes to the tree topology, a number of portions of the tree will remain identical. Therefore, it is likely that the optimal values of subtree branch lengths will not change. It is, therefore, helpful to represent branch lengths in a method that is robust to these changes to avoid carrying out the full optimization process every time the topology is changed.

Within the Phylo2Vec framework, there are several approaches in which branch lengths can be integrated. First, given each $v_{j}$ refers to the branch splitting and leading to leaf j, a simple solution would consist in adding a two-column matrix that specifies the position at which branch $v_{j}$ splits and the length of the new branch yielding leaf j. Alternatively, it is possible to assign each leaf node a “position,” calculate internal node positions as some weighted average of the positions of the nearby leaf nodes, and then calculate branch lengths based on the distance between a pair of nodes. This would have the advantage of branch lengths being independent of the choice of root, thus allowing to easily switch between the unrooted equivalence classes discussed previously.

For the examples in this paper, we used RAxML-NG (Kozlov et al. 2019) to optimize the branch lengths at each step of the algorithm without using information from previous branch lengths. This reduces the speed of the optimization and is an area for improvement in future work.

### Evaluation

#### Problem and data

To demonstrate the utility of Phylo2Vec, we apply our new representation for phylogenetic inference of five popular empirical molecular sequence datasets under the ML criterion. This dataset corpus spans across different biological entities, taxa, and genetic sequence sizes.

It has been proved that ML inference for phylogenetic trees is NP-hard (Roch 2006) and therefore our key goal is to define a sensible heuristic that can explore the vastness of tree space. Moreover, the likelihood surface exhibits high curvature (Sanderson et al. 2011) and being trapped in a local optima is a persistent problem across all heuristic phylogenetic approaches.

#### Tree topology optimisation using hill-climbing

A simple way to explore the space of possible trees is to use hill climbing where we simply compute the difference in likelihood after a single element is changed. We define the neighbor matrix


$$\begin{array}{c} \Delta \ell {\left(v\right)}_{ij}=\ell ((v_{1},\ldots ,v_{i-1},j,v_{i+1},\ldots ,v_{n-1}))-\ell (v), \end{array}$$ (4)

that is, the tree considered in the first likelihood has identical entries except for the $i^{\text{th}}$ entry, which is changed to j. For (i,j) such that $v_{i}=j$ is infeasible, we set $\Delta \ell_{ij}=0$. We have found that considering each row of the neighbor matrix yields good results, that is, if $\max(\Delta \ell_{i})>0$, then we find $j={\text{argmax}}_{j}(\Delta \ell_{ij})$ and change the value of $v_{i}$ to j. This algorithm is guaranteed to converge to a point where  max ⁡(Δℓ)≤0 as no change in v results in a gradient that is greater than zero. Moreover, as there are only finitely many possible v, and ℓ is strictly decreasing after each iteration of the while loop, the algorithm must converge in finite time. More complicated optimization algorithms can be readily created and is an especially useful aspect of our representation. An example is performing hill-climbing over paired changes in v. Exploratory analysis suggests that paired changes are far more robust to being trapped in local minima, but at the cost of higher complexity. For challenging phylogenies, a simpler parsimony or minimum evolution score can be used to perform hill-climbing over pairs as an exploratory search.

However, as highlighted above, a fundamental asymmetry exists in Phylo2Vec that can make optimization inefficient. A simple solution to mitigating this asymmetry is to reorder the integer-taxon mapping to obtain an ordered vector (and thus, an ordered tree), as described previously in “ An Incomplete Integer Representation of Trees as Birth Processes” section and Figure 6. The advantage of carrying out our hill climbing scheme on these ordered trees is that it removes the secondary effects of changing an element of v which can occur by the divergence in internal node labels. This prevents our algorithm from getting stuck in local minima, as it means that more parts of the tree can be easily edited.

The resulting algorithm is detailed in Algorithm 1. Our investigations have shown that all the possible trees that are one step from some ordered v are also one SPR move from the original tree (though the converse is not true—not all SPR moves will be one step from v). This is proved in the Appendix (Phylo2Vec details). Thus, this application of our Phylo2Vec formulation falls within the SPR framework and provides a mathematically convenient and principled way to explore tree space using well-tested SPR methodology.

We note that we could additionally explore rooted equivalence classes to further prevent being stuck in local minima. In particular, there is more freedom in the movements of nodes further down the tree, and re-rooting at the deepest node would allow all nodes to be easily moved to a variety of locations. However, for the experiments presented hereinafter, we found this extra degree of freedom to be unnecessary.

**Table:** 

Algorithm 1: Hill-climbing optimization of a tree with n leaves	
**Input** $v\in T_{n}$	▹ Initialize with a random v
$\ell_{\text{best}}\leftarrow \ell (v)$	▹ Initial best likelihood value
**Repeat** Reorder(v)	▹ Reorder the labels (see Fig. 6)
Sample i∈{1,…,n-1}	▹ Sample an index of v
$G_{i}\leftarrow \Delta \ell {\left(v\right)}_{ij}$	▹ $G_{ij}$ = likelihood difference from changing $v_{i}$
to j	
$j\leftarrow \text{argmax}(G_{i})$	▹ Find the best change
$v_{i}\to j$	▹ Change $v_{i}$
$\ell_{\text{best}}\leftarrow \ell (v)$	
**Until** $\max(G_{i=1,\ldots ,n-1})=0$	▹ Continue iterating until local minimum

#### Additional properties of the Phylo2Vec vector

**Figure 7:**
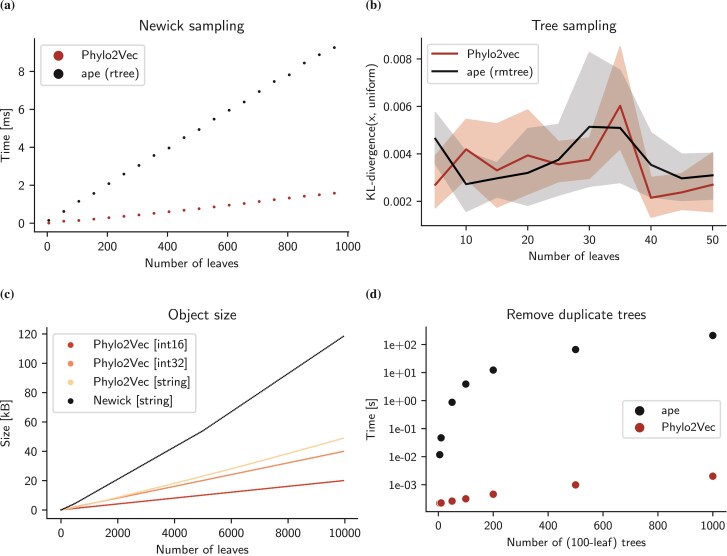
Phylo2Vec allows for fast and unbiased sampling, low memory or storage, and fast comparison of trees. a) Average sampling time using phylo2vec.utils.sample and rtree from ape. Execution time was measured over 100 executions using Python’s timeit and R’s microbenchmark, respectively. b) Sampling bias comparison. For each size and sampler, we sampled 10,000 trees and converted them first to their Phylo2Vec representation and second to an integer using a method similar to that of Rohlf (1983). We then compare the probability distributions of the integers generated by Phylo2Vec and ape sampling against the reference uniform distribution for each tree size using the Kullback–Leibler (KL) divergence. The lower the KL-divergence value, the more the reference distribution and the distribution of interest share similar information. c) Object sizes for different tree sizes of Phylo2Vec vectors (stored as a 16- or 32-bit numpy integer array, or a string) compared against their Newick-format equivalents (without branch length information). d) Average time for duplicate removal from a set of trees using Phylo2Vec (vectors) and the *unique.multiPhylo* function from ape. Execution time was measured over 30 executions using Python’s timeit and R’s microbenchmark, respectively.

An additional advantage of having an integer vector representation for binary trees such as Phylo2vec is efficiency with respect to sampling, data storage, as well as assessing tree equality (with respect to topology). We highlight these properties in Figure 7 by performing several benchmarks against functions of shows the widely used R library apeParadis and Schliep (2019). Figure 7a shows how Phylo2Vec sampling of trees is several times faster than the function rtree, while also being simple in construction and implementation. Figure 7b verifies that the Phylo2Vec sampling distribution is indeed uniform. While we do explore other sampling schemes further, ordered trees present one avenue to perform constrained tree sampling. Figure 7c shows the storage costs in kB of Phylo2Vec as compared to a Newick string with *only* topological information. From these simple simulations, we estimate a Phylo2Vec vector can be stored as an integer array or a string as much as a six times reduced storage cost. Finally, Figure 7d shows the time required to find a unique set of topologies from a set of trees. Phylo2Vec is several orders of magnitude faster than unique.multiPhylo in ape, and can be massively parallelized. This speed difference can be particularly useful in Bayesian settings.

### Implementation

All Phylo2Vec algorithms and related optimization methods presented in the main text were implemented in Python 3.10 using NumPy (Harris et al. 2020) and numba (Lam et al. 2015). Tree manipulation scripts were written using ete3 (Huerta-Cepas et al. 2016). Dataset construction was based on phangorn (Schliep 2011) in R and TreeTime (Sagulenko et al. 2018) in Python. ML estimation was performed using RAxML-NG (Kozlov et al. 2019). An implementation is available at: https://github.com/Neclow/phylo2vec. Execution times were benchmarked using Python’s timeit on a machine equipped with a 64-core CPU @ 7 GHz, with 256 GB of RAM.

## Results

We test Phylo2Vec by performing inference on five popular empirical datasets described in Table 1. This dataset corpus spans across different biological entities, taxa, and genetic sequence sizes.

**Table 1:** Evaluation datasets, sorted by number of taxa.

Name	Reference	Type	# taxa	# bases
Yeast	Rokas et al. (2003); Schliep (2011)	Fungi	8	127,018
H3N2	Sagulenko et al. (2018)	Virus	19	1407
M501	Garey et al. (1996)	Animal	29	2520
FluA	Fourment and Darling (2019)	Virus	69	987
Zika	Sagulenko et al. (2018)	Virus	86	10,807

For each dataset, we use the optimization procedure described in Evaluation, using RAxML-NG for branch length and substitution matrix optimization. We report performance using the negative log version of the tree likelihood defined by Felsenstein (1983).

**Figure 8:**
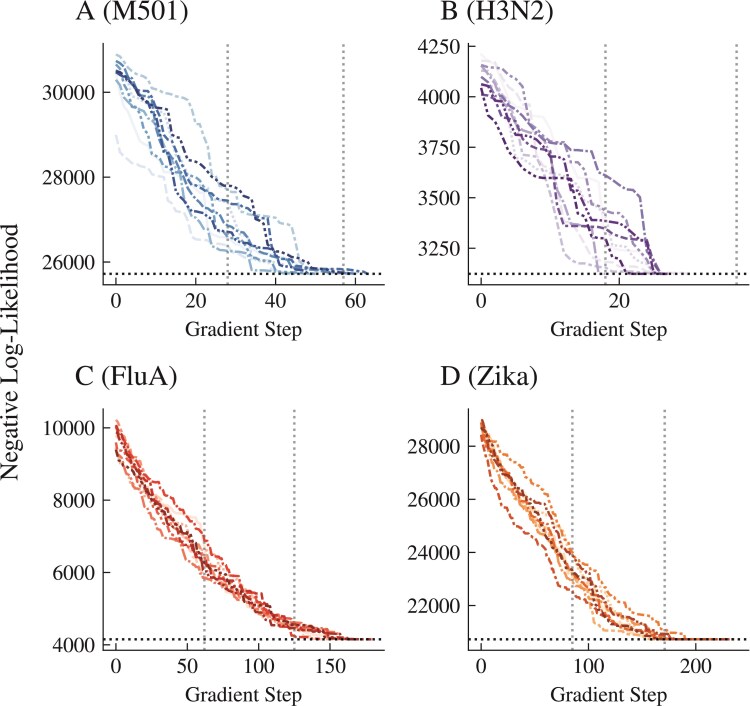
Phylo2Vec-based likelihood optimization results for 4 datasets are described in Table 1. The horizontal and vertical lines indicate local minima and epochs (i.e., one pass through every index of v), respectively.

Figure 8 shows the optimization results for four of the datasets described in Table 1. We observe that from ten random starting trees, we always achieve the same minimal loss without being trapped in local optima. This is comparable to state-of-the-art software that also searches through topological space (Stamatakis 2014; Minh et al. 2020). For each dataset, only two epochs of changes (that is, 2 passes through every index of v) were generally needed to achieve a minimal negative log-likelihood. In addition, for M501, for example, only a total of around 10,000 likelihood optimizations for each run were needed to reach a minimum—a vanishingly small fraction of the total number of trees possible with 29 taxa (~8${\text{e}}^{36}$). The choice of the number of optimizations can be shortened depending on the optimization stoppage criteria, but with the trade-off of being trapped in local minima. We also note that in the Zika virus example, two runs converged at a loss slightly (0.07%) greater than the minimum of the other eight runs. The resultant trees from these minima show that we get trapped in these suboptimal minima due to rooting issues, preventing single changes in v from finding a better optimum. This highlights once again that our algorithm is attempting to solve a more difficult problem than is strictly necessary by searching the space of rooted trees rather than unrooted trees. Due to the pulley principle (Felsenstein 2004), all rootings of an unrooted tree have the same negative log-likelihood and, therefore, no paths between rooted trees exist to aid our optimization algorithm. In practice, especially for large phylogenies, it is common to begin optimization from a sensible starting point (Paradis et al. 2004) (e.g., a maximum parsimony or a neighbor joining tree). In our experiments, we have chosen to start from a completely random tree to highlight the utility of simple algorithms based on Phylo2Vec to traverse tree space.

**Figure 9:**
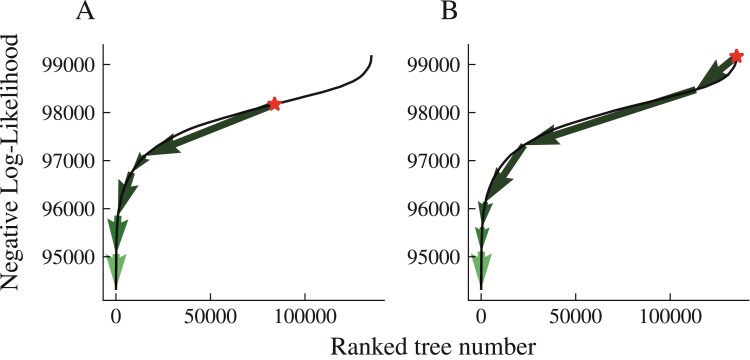
A negative log-likelihood path drawn from all possible trees of the Yeast dataset. A and B, respectively, show the path to the minimum from a random tree and the worst possible tree. The solid line shows the sorted phylogenetic likelihoods for all trees. The arrows show the proposal moves for 2 searches, one from a random tree ) and one from the worst possible tree B).

Subsequently, we apply the same optimization procedure for the yeast dataset (eight taxa) initially presented in Rokas et al. (2003) and studied in Money and Whelan (2012). Given the smaller number of taxa, we were able to exhaustively calculate the likelihood for every possible rooted tree. As shown in Figure 9, we notice a broad region of numerous trees with comparable likelihoods, in addition to a considerably smaller group of trees exhibiting increasing likelihood. Regardless of whether we start from a random tree or the worst possible tree, our algorithm quickly converges to the accurate tree reported in Rokas et al. (2003). Across several runs, Algorithm 1 required 96 total likelihood evaluations—a very small fraction of the total number of trees.

## Discussion

Phylo2Vec is a parsimonious representation for phylogenetic trees whose validity extends to any binary tree. This representation facilitates the calculation of distances between trees and allows the construction of a simple algorithm for phylogenetic optimisation. Following from trends in phylogenetics, Phylo2Vec could be integrated within state-of-the-art computing libraries (e.g., libpll (Flouri et al. 2015) or Beagle (Ayres et al. 2012)) to facilitate its use. We have not yet considered Bayesian inference, but this is likely a useful application of Phylo2Vec, where random walks can be trivially implemented (see Fig. 5). Furthermore, Phylo2Vec can be useful in assessing topological convergence, for example, for a large phylogeny of 500 taxa and a million trees, extracting the unique set of topologies takes <10 s on a single core in Python, and can be even faster with parallel computation. Although Phylo2Vec does allow for unrooted trees, it is primarily an algorithm for rooted trees. In the examples in this paper, we only consider reversible Markov models where rooting is irrelevant due to the pulley principle (Felsenstein 2004). Irreversible Markov models are both mathematically and biologically more principled (Sumner et al. 2012) but require rooted trees. Therefore, a useful application of Phylo2Vec could be in the inference of phylogenies with irreversible Markov models.

The use of empirical datasets served as a proof of concept that ML estimation can be performed using Phylo2Vec vectors. We show that, using a simple hill-climbing scheme, we can recover the same topology optimum found by state-of-the-art MLE frameworks such as RAxML-NG (Kozlov et al. 2019). It is important to note, however, that this approach is nowhere near as optimized as RAxML-NG. As it only performs topology changes at a single vector index at a time, its inherent greediness makes inference of large datasets difficult.

That being said, the simplicity of the Phylo2Vec formulation means that it can be used in other more efficient and complex optimisation schemes can also be developed. For instance, Phylo2Vec can also benefit from fast SPR changes (Guindon et al. 2010) and other heuristic optimizations that are currently in RAxML (-NG). In addition, by construction, we have ensured that Phylo2Vec can be differentiable through transforming v into a matrix $W\in {\mathbb{R}}^{0,1}$ such that $W_{ij}=\mathbb{P}(v_{i}=j)$. Via this transform, inference in a continuous tree space using gradient descent-based optimization frameworks is theoretically possible, but its particulars remain to be developed. Similarly, we expect Phlyo2Vec-based representations to be applied in Monte Carlo tree search (MCTS) frameworks that may explore tree space more efficiently, or used as an embedding to regularly infer phylogenetic trees using well-established machine learning paradigms such as self-supervised learning from large existing tree libraries (e.g., TreeBase (Piel et al. 2009)).

## Author contributions

S.B., N.S., and M.J.P. conceived the study. S.B. supervised. S.B., N.S., and M.J.P. designed the study. S.B., M.J.P., and N.S. performed optimization runs. S.B., M.J.P., and N.S. performed analysis. S.B., M.J.P., and N.S. drafted the first original draft. All authors contributed to editing the original draft. N.S. and D.A.D. contributed to revisions of the methodology. M.J.P., N.S., and S.B. drafted the appendix.

## Supplementary Material

Supplementary data is available at *SYSBIO* online.

## Conflict of interests

None declared.

## Funding

M.J.P. acknowledges support from his EPSRC DTP studentship, awarded by the University of Oxford to fund his DPhil in Statistics. D.A.D. acknowledges support from the Novo Nordisk Foundation via The Data Science Investigator Award (NNF23OC0084647). S.B. acknowledges funding from the MRC Centre for Global Infectious Disease Analysis (reference MR/X020258/1), funded by the UK Medical Research Council (MRC). This UK-funded award is carried out in the frame of the Global Health EDCTP3 Joint Undertaking. S.B. is funded by the National Institute for Health and Care Research (NIHR) Health Protection Research Unit in Modelling and Health Economics, a partnership between UK Health Security Agency, Imperial College London and LSHTM (grant code NIHR200908). The views expressed are those of the author(s) and not necessarily those of the NIHR, UK Health Security Agency or the Department of Health and Social Care. S.B. acknowledges support from the Novo Nordisk Foundation via The Novo Nordisk Young Investigator Award (NNF20OC0059309). S.B. acknowledges support from the Danish National Research Foundation via a chair grant (DNRF160), which also supports N.S. S.B. acknowledges support from The Eric and Wendy Schmidt Fund For Strategic Innovation via the Schmidt Polymath Award (G-22-63345). S.B. and N.S. acknowledge the Pioneer Centre for AI, DNRF grant number P1 as affiliate researchers. C.A.D. receives support from the NIHR HPRU in Emerging and Zoonotic Infections, a partnership between the UK Health Security Agency, University of Liverpool, University of Oxford and Liverpool School of Tropical Medicine (grant code NIHR200907).

## Data availability

All code relevant to reproduce the experiments is available online: https://github.com/Neclow/phylo2vec. Instructions to access the publicly available datasets used in this study are included in the phylo2vec/datasets folder of the repository.

## Appendix

In this appendix, we present a rigorous mathematical framework summarizing the results in this paper. We also detail further algorithms that may be useful in the implementation of Phylo2Vec.

### Notations and Definitions

#### Notations

We shall use the notation T to refer to a tree, b for the branch lengths of T and n as the number of leaves (or taxa).

#### Node labels

In a tree with n leaves, we set the convention that the leaf nodes are *labeled* from 0 to n-1, the internal nodes are *labeled* from n to 2n-3 and the root (if it exists) is labeled as 2n-2.

#### Generation

By definition, there is a unique path connecting each pair of nodes in a tree. Suppose that the path from node i to the root contains the nodes $i,a_{1},\ldots ,a_{g_{i}}$ in that order (and the root is hence $a_{g_{i}}$). We call $g_{i}$ the *generation* of node i. The path from any $a_{x}$ to the root must be $a_{x},a_{x+1},\ldots ,a_{g_{i}}$ and hence $g_{a_{x}}=g_{i}-x$.

#### Unrooting

Suppose that, in a rooted tree, the 2 children of the root are x and y. To “unroot” the tree, we remove the edges connecting x and y to the root and add a new edge connecting x and y. This edge is given length $b_{x}+b_{y}$ where, for example, $b_{x}$ is the length of the original edge joining x to the root. We shall use $T^{\prime }$ to refer to the unrooted tree and $b^{\prime }$ to its branch lengths.

### Phylo2Vec details

#### Vector representation

As described in the main text, Phylo2Vec is a way to represent binary trees with n leaves using a single integer vector, v of dimension n-1 that is simply constrained by

v[j]∈{0,1,…,2(j-1)}∀ ⁡j∈{1,…,n-1}

The construction of the tree from this representation and the bijectivity between v and the space of trees are covered in the main text.

#### Bijectivity of v tothe space of all possible trees

We can show that our mapping from V to the space of trees is a bijection.

*Lemma 1. The mapping between the set of vectors v∈V and the set of (topologically equivalent, labeled) trees is a bijection.*

**Proof:** As, by construction, the number of possible vectors v and the number of trees are the same ((2n-3)!!), it is simply necessary to show that this mapping is injective.

For any two nodes a and b, we define M(a,b) to be their most recent common ancestor (MRCA). Moreover, for any nodes a and b, we use the notation a≺b if a is an ancestor of b.

We can use the fact that the MRCA of any pair of leaf nodes in the tree is unchanged during the Phylo2Vec construction process (once both nodes have been added to the tree). Note that the path from a node x to the root changes through the addition of an extra node if and only if a new node is appended to an edge on this path. Thus, if M(a,b)=x, x will remain on the paths from a and b to the root. Moreover, nodes of higher generation than x can only be added to one of the paths (as otherwise there would have to be an edge connecting at least one node of higher generation than x on both the original paths, contradicting x being the MRCA). Thus, the MRCA of a and b will be unchanged. Similarly, we can see that a≺b for two a and b at a given stage of the algorithm, this relationship will be unchanged throughout the construction process.

Suppose that 2 vectors v and $v^{\prime }$ result in trees T and $T^{\prime }$, and that $v\neq v^{\prime }$. Define $i:=\min\{j:v_{j}\neq v_{j}^{\prime }\}$.

Define X to be the set of leaf nodes that, just before node i is added, are descended from the edge to which node i is appended when constructing the tree according to v, and $X^{\prime }$ to be the equivalent set for $v^{\prime }$. We have $X\neq X^{\prime }$, as each edge has a unique set of nodes descended from it.

If both $X\backslash X^{\prime }$ and $X^{\prime }\backslash X$ are non-empty, suppose that $a\in X\backslash X^{\prime }$ and $b\in X^{\prime }\backslash X$. Then, in T, it must be the case that M(b,i)≺M(a,i) (both at this stage in the algorithm, and in the final tree, by the previous argument) once node i has been added (as the M(a,i) will be the new internal node and M(i,b) cannot be either i or this new internal node). A similar argument shows that M(a,i)≺M(b,i) in $T^{\prime }$ and hence $T\neq T^{\prime }$.

If only one of $X\backslash X^{\prime }$ and $X^{\prime }\backslash X$ is non-empty, suppose without loss of generality that $X\backslash X^{\prime }$ is non-empty and choose $a\in X\backslash X^{\prime }$. We can choose a distinct node $b\in X^{\prime }\cap X$ (as otherwise, if $X^{\prime }\cap X$ and $X^{\prime }\backslash X$ are both empty, we must have one of X and $X^{\prime }$ being empty which is a contradiction as every edge has at least one leaf node descended from it). Then, M(i,a) is the newly added internal node in the construction of T, but not in the construction of $T^{\prime }$. However, in both cases, M(i,b) is the newly added node. Hence, in T, M(i,a)=M(i,b), but in $T^{\prime }$, they are distinct. Thus, $T^{\prime }\neq T$.

Hence, topological non-equivalence holds in both cases, and the map from v to the set of trees is, therefore, injective and, therefore, bijective.

#### Label-asymmetry of v-induceddistance

As discussed in the main text, v induces a natural distance function between trees—namely, that the distance between v and w is equal to $\sum_{i=0}^{k}I_{v_{i}\neq w_{i}}$.

However, this distance function is dependent on the labels assigned to each leaf (and is hence label-asymmetric). A simple example of this can be found in the case of 4 leaves.

Consider the tree given by $v_{1}=(0,1,2)$. This is a ladder tree (that is, each internal node and the root is parent to at least one leaf node) with nodes in order 0,1,{2,3} (where the {2,3} is used to denote the fact that nodes 2 and 3 are from the same generation and so could be read in either order). This is distance 1 away from $v_{2}=(0,1,4)$, which is again a ladder tree with nodes now ordered as 3,0,{1,2}. Thus, if distance were symmetric, then any ladder tree with ordered nodes a,b,{c,d} would be distance 1 away from the ladder trees with ordered nodes d,a,{b,c} and c,a,{b,d}. However, the ladder tree with ordered nodes 2,0,{1,3} is given by $v_{3}=(0,2,1)$, which is distance 2 away from $v_{1}$. Thus, the distance function is not label-symmetric.

#### Unrooted tree equivalence classes

Noting from the main text that there are $\left(2k-3)!!=\prod_{i=0}^{k-2}(2i+1\right)$ unrooted trees with k+1 leaves, we can partition the space of trees with k+1 leaves into (2k-3)!! equivalence classes $\left\{E_{i}:i=1,2,\ldots ,(2k-3)!!\right\}$ such that the removing the root from each of the trees in a given class $E_{i}$ (according to the procedure described in Notations and Definitions in the Appendix) results in the same unrooted tree. These equivalence classes all contain (2k-1) trees (as, reversing the unrooting procedure, a root can be added onto each of the (2k-1) edges of an unrooted tree).

From the previous work, Felsenstein’s likelihood is constant in each equivalence class. This has 2 consequences when attempting to maximize the likelihood. First, a global minimum in the space of rooted trees will not be unique, as any of the other trees in the same equivalence class will also give a maximal likelihood. Second, if v is a local maximum (i.e., all w with one entry different to v give a lower likelihood value), equivalence classes provide a way to change to a new vector u without having to decrease the likelihood.

Similarly to the case of reordering, the set of equivalence classes of vectors that are distance 1 from v will not, in general, be the same as the set with distance 1 from u. One can either change equivalence class randomly or (unlike in the case of reordering), as there are only 2k-1 members of each class, systematically iterate through them. Finding the vectors w corresponding to the trees in an equivalence class can again be done by constructing the tree matrix M, making the appropriate changes and then using the inversion algorithm to create a new vector representation.

Our experiments have shown that changing v is effective in increasing algorithmic performance. However, we found that reordering provided better increase in performance, and, therefore, did not present this other switching method in the algorithm in the main text.

#### Number of moves

Let a vector v of length n-1 representing a tree with n leaves. For each entry j, there are 2j-1-1=2(j-1) possible moves, as there are 2j-1 possible entries for entry j, but v[j] is already set). Summing for all j∈1,…,n-1 gives:

$$\overset{n-1}{\underset{j=1}{\sum }}2(j-1)=(n-1)(n-2)\approx n^{2}$$

Thus, the number of possible moves for Phylo2Vec is of the order $O(n^{2})$.

### Algorithms

The algorithms provided here are presented in rough descriptive pseudocode; they are written in human-readable fashion to facilitate comprehension, but are not necessarily optimized. Please refer to the GitHub repository (https://github.com/Neclow/phylo2vec) for detailed implementation.

**Table:**

Algorithm S1 Labeling a rooted tree as an ordered v	
**Input** rooted tree T with n leaves	
v←[0]	⊳ Initial integer vector
**for all** leaves j=2,...,n-1 **do**	
$T_{j}\leftarrow$ subtree with leaves 0,...,j	
s← leaf in $T_{j}$ such that j and s form a cherry	
v.append(s)	
**end** **for**	
**return**v	

**Table:**

Algorithm S2 Recovering an ordered rooted tree from an ordered v	
**Input**v of size n-1 satisfying Equation (1)	⊳ Ordered integer vector
T← a rooted tree with two leaves 0 and 1	⊳ Initial tree
**for all** j=2,...,n-1 **do**	
Add a new leaf j to T such that it forms a cherry with v[j]	
a (j, v[j]) ←2(n-1)-j+1	⊳ Ancestor of j and v[j]
**end** **for**	
**return**T	

**Table:**

Algorithm S3 Recovering a rooted tree from a Phylo2Vec v	
**Input**v of dimension n-1 satisfying Equation (2)	⊳ Phylo2Vec vector
pairs ←[(0,1)]	⊳ The objective of the algorithm is find the nodes to merge and their order. The first pair is always (0,1) for a 2-leaf tree.
**for all** leaves j=2,...,n-1 **do**	
**if** v[j]≤j **then**	⊳ This is like an ordered vector. The branch leading to v[j] gives birth birth to leaf j
next pair ←(v[j],j)	
position ← 1	⊳ This pair corresponds to a cherry at height 0, so it should be drawn first.
**else**	⊳ The branch to be split is internal
position ←v[j] - len(pairs) + 1	⊳ The index at which the next pair will be inserted
descendant ← pairs[position - 1][1]	⊳ A node that we processed beforehand that is deeper than the branch v[j]
next pair ← (pairs[descendant, j])	
**end** **if**	
pairs.insert(position, next pair)	
**end** **for**	
**return** pairs	⊳ The pairs indicate how to build the tree (and form a Newick string)

**Table:**

Algorithm S4 Labeling a rooted tree as a Phylo2Vec vector	
**Input** rooted tree r with n leaves	⊳ We assume a Newick string with integer nodes and labeled internal nodes. Ex: (((2,3)6,1)7,(0,4)5)8;
M← reduce(r)	⊳ “Reduce" the Newick string to a (n-1)×3 matrix. Columns 1-2 = children nodes Column 3 = ancestor Ex: $\begin{array}{ccc} 0 & 4 & 5\\ 2 & 3 & 6\\ 1 & 6 & 7\\ 5 & 7 & 8 \end{array}$
C← extract_cherries(M)	⊳ Starting from the smallest internal node, replace the internal nodes by their smallest child (and discard the third column). This is equivalent to the pairs in Algorithm S3 Ex: $\begin{array}{cc} 0 & 4\\ 2 & 3\\ 1 & 2\\ 0 & 1 \end{array}$
v← build_vector(C)	⊳ Use the same logic as Algorithm S3 to retrieve each $v_{j}$ from the position of the containing leaf j
**return**v	
